# Editorial

**Published:** 1875-05

**Authors:** 


### Editorial.

### TREATMENT OF EXPOSED TOOTH PULP.

This is a subject of great importance to every dentist, and one of vital importance to every person who may be afflicted with such exposure. It is a matter of life and death; in it rests the preservation or loss of the tooth or teeth involved.

Until within a few years the treatment of exposed pulp was very inefficient with the great majority of dental practitioners.

Indeed none were as successful as a large portion of the profession now is, by the use of the present knowledge upon the subject. The principles of proper treatment are now well understood; still, in the minds of some, there seems to be a vagueness of apprehension of these principles that is surprising. This arises, chiefly perhaps, from an indisposition to study principles and to engage in thorough analytical investigation, and a desire to have recipes, formulas and specifics. These are much easier to understand and apply than principles.

We have upon various occasions endeavored to present these principles as definitely and clearly as we could, and quite as often given what we regard as the correct mode of treatment, and the materials and agents best adapted for the purpose.

In the treatment of any deranged or diseased organ or tissue of the body it is desirable and important to understand its structure, function and susceptibilities, also life endowment and resisting and recuperative power; and, in addition to this, a thorough understanding of the phases of disease. When these points are all attained, then treatment with its appropriate agents comes legitimately, intelligently and efficiently. Exposed and diseased pulps of teeth are no more susceptible to a panacea, cure all, or single mode of treatment than any other tissue or structure of the body, and vain has been the search for an agent or method of universal application and efficiency. The great desire with many is to know the particular course to be pursued or the thing to be done and the agent to be used, and, that too often, without any attempt or wish to possess the knowledge of conditions that should modify and govern the particluar steps in treatment. In the treatment of exposed tooth pulp, there are some things common to almost all cases. And in the first place it is in every instance necessary to remove every irritant from the tissue so far as possible. The method and facility of accomplishing this will be governed by the character of the irritants, and the susceptibilty of the pulp to irritation. All irritating agents should not only be removod but permanently excluded. This applies to all cases where the pulp has been exposed by decay of the teeth. The materials that are usually offensive are the debris of decaying dentine, the foreign substances that accumulate and undergo decomposition in the cavities of decay, and vitiated exudation from the pulp.

The first two of these may be removed by excavation, and thorough washing with water, and sometimes in addition a mild disinfectant and antiseptic will be required.

All injurious agents must not only be removed, but permanently excluded; for, according to nature's plan, the pulps of the teeth were never to be brought into contact with anything except the walls of their own chambers. Vitiated and offensive exudation will oftentimes proceed from diseased pulps, even after the orifice of exposure has been closed; it will not only act as an irritant while the cavity of decay is open, but after it is closed as well, as is shown by the offensive odor sometimes found in a cavity that has been hermetically sealed, and a living pulp beneath. And in other cases it is found that a thin layer of undecomposed dentine is perforated after the cavity of decay has been filled so as to exclude any foreign substance, such perforation is effected by a vitiated product of an irritated pulp. Such cases can not always be anticipated. But when a pulp is already exposed it is usually amenable to treatment and restoration to such an extent as to perform its normal function. Every one, intelligent in this matter, will recognize at once that there is a very great variety of susceptibility in different cases, and even in the same case at different times and in different conditions. The restoration of an exposed pulp to a healthy condition is the second step in the order of its treatment.

The question, of course here occurs, can this always be done? And what is the proper treatment for such restoration? In answer to the first question, it can not always be done; but in the large majority of cases it can; the proportion will vary in different localities. Success, with even the best treatment, that which is according to the highest present attainments in this direction, will be modified by the resisting and recuperative life force in the organ, and by its condition when treatment is begun, by the nutrient supply it receives through the system; and by the size, form and location of the orifice at which it is exposed; these governing the facility of approach and manipulation.

A pulp in a state of irritation and inflammation, like any other ordinary tissue in similar conditions, has in it and passing through it an excess of blood, and that too, changed from its normal condition, just as in other tissues or organs. The vessels then become subject to the same vicisitudes as elsewhere, and require the same relief. The nerve structure of the pulp is unduly impigned or pressed upon, and in addition fails to receive its proper nourishment and, in many cases, is undoubtedly affected by poisonous matter with which it comes in contact, either that from without, or that elaborated in the structure; and the great outcry, so often experienced, is the result.

The circulation in the structure is to a greater or less extent retarded or arrested. The blood becomes surcharged with waste matter and is deficient in oxygen. Now the demand here is restoration of the circulation to a normal condition, and thereby giving tone and vigor to the relaxed and feeble vessels, and relieving the nerves of their embarrassment. The proper management of the circulation will usually relieve the difficulty. Draw away the excess, this relieves distension and pressure—the active flow brings fresh blood freighted with nutrient material which imparts tone, strength and activity to the vessels; and debris and effete matter will be caught up and eliminated and the entire structure returned to a normal activity.

The best results from an attempt at restoration to a normal circulation in an inflamed pulp will and more easily attained where the organ is freed from all unfavorable influences. Usually after removing the foreign substances from the surface of an exposed pulp, the exposed surface scarcely presents the appearance of living tissue and indeed has become so much impaired that its removal is a necessity, if the body of the organ is to be preserved and restored to health. This removal may be effected by excision, as is often done. This method was first presented definitely to the profession by Dr. Allport, who clearly demonstrated that a portion of tooth pulp can be removed and the remaining part restored to a healthy condition. This may be done even to the removal of one-half of the pulp. About the only cases in which excision seems to be indicated, are those in which there is protrusion of the pulp through the orifice of exposure.

In the majority of cases another method is preferable, this is by the use of some agent that will dissolve any debris or partially devitalized tissue that is not susceptible of restoration. The use of pepsin in the treatment of exposed pulp was brought to the notice of the profession about four years ago, by Dr. Oakley Cales, of London.

It was simply prepared and used without any clear definition or even theory as to its mode of action, it was only stated that experiment had decided that it was valuable in many cases. It is good and its efficiency consists in its ability to dissolve any debris that may be in contact with the exposed pulp, and devitalized or even partially devitalized or dying pulp tissues; the living active tissue resists its action almost entirely if not altogether. After a paste of pepsin, composed of the liquid and dry pepsin, has been in contact with an exposed pulp for from four to six hours, a solution of the debris will have taken place, the pulp will present a clean surface and natural color. The same result may be produced by the use of diluted hydrochloric and lactic acids mixed with a proper vehicle, phosphate of lime for instance, or indeed any substance of such a character may be used, between which and the acids there would be no injurious action.

Lacto-phosphate of lime, and lactic acid and phosphate of lime have been used for the last year and a half, with very decided success in many cases. The mode of its action, and the work it accomplishes is of the same nature as that performed by the agent refered to above. Though it has been suggested that the phosphate of lime is taken up and appropriated, and aids in the elaboration of secondary dentine. This theory however, so far as we are aware is purely hypothetical. To Dr. J. E. Cravens, of Kansas City, Mo., is due the credit of proving the value of this agent and of introducing it to the profession.

In the great majority of cases the treatment already indicated, together with due attention to systemic conditions will restore the exposed and diseased pulp to that condition in which it may be with safely enclosed. Many methods of covering or capping exposed pulps have been suggested, and practiced. The requirement in all instances is to inclose it as nearly after nature's plan as possible; and in order to do this the material used to cover it should be one that could be readily applied, so as to occupy all the space and yet without making pressure upon the pulp or producing any irritation; a material that will not undergo decomposition or change in this position, and will be sufficiently resistant to admit the cavity of decay to be filled with gold, or any desirable material; it should also not transmit thermal change more readily than dentine. Now these requirements are easily understood; but the substance to answer them completely, is not yet obtained. There are however materials that, in a good degree serve the purpose. The substances hitherto employed are so well known to the profession that it seems unnecessary to mention them here. Oxy-chloride of zinc has been thoroughly tried, but failures occurred so often when it alone was relied upon that it has been almost if not entirely abandoned for this purpose; the free chlorine that is always present when the preparation is of a consistence to be used, generally proves an active and dangerous irritant, frequently resulting in the death of the pulp to which it is applied. Moistening the exposed part with carbolic acid before the application will somewhat lessen the evil. A better method than this, is that suggested by Dr. King a few years ago, and published in REGISTER at the time, which was leaving out the chloride and making a paste of the oxide of zinc and creosote or carbolic acid, and with this fill and cover the orifice of exposure, then remove any excess of creosote with bibulous paper, then over this place the oxy-chloride which serves as a protection to the paste, and forms a solid foundation for the reception of the filling.

The lacto-phosphate of lime in paste form is used by some for filling and covering the orifice of exposure; and with quite as good if not better results than with the oxide of zinc and creosote. From some recent experiments the lacto-phosphate would seem to be preferable. It is highly probable that a material better than either of them will be found ere long, but they are a great way in advance of any thing before used.

---

### EASTERN INDIANA DENTAL ASSOCIATION.

The semi-annual meeting of the Eastern Indiana Dental Association, will be held at Dublin, Indiana, Tuesday and Wednesday, May 4th and 5th, '75.

PROGRAMME OF, SUBJECTS FOR DISCUSSION:

1. Duties of the Dentist.
2. Idiosyncrasies of the patient to be considered, as to failure or success in filling teeth.
3. Systemic conditions, and modes in treatment of pulpless teeth.
4. Sensitive dentine and pain obtunder.
5. Cohesive or non-cohesive gold—should we use one, to the exclusion of the other?
6. Almalgam—should we use it? If so, when and how?
7. Instruments and appliances, use and relative merit.
8. Mechanical dentistry, its status.
9. Code of Ethics.

Clinics from 8 A. M. to 12 M., Wednesday, May 5th.

Volunteer Essay solicited on any subject to advance the interest of the profession. Every member requested to aid the discussions by essays or thought on the subjects enumerated.

N. B. Dentists not members, and Physicians are respectfully invited to attend our sesssions.

[Meet at Dr. Stanley's Office, at 9 A. M., May 4th.]

President J. K. JAMISON, Shelbyville,

### ILLINOIS STATE DENTAL SOCIETY.

The eleventh annual meeting will be at Ottawa, May 11th.

ORDER OF BUSINESS.

TUESDAY, MAY 11TH—MORNING SESSION.

1. Call to order, roll-call and reading of minutes.
2. Applications for membership.
3. Reports of Committees.
4. Reports of Officers.
5. Election of members.
6. Miscellaneous business.
7. Adjournment.

AFTERNOON SESSION.

1. Annual Address by the President.
2. Essays and Discussions.

EVENING SESSION.

Essays and Discussions.

WEDNESDAY, MAY 12TH—MORNING SESSION.

This session will be principly or entirely devoted to clinics, to be conducted by Drs. G. H. Cushing, Chicago; W. T. Smith, Peoria; W. DeCrow, Quincy; S. C. Wilson, Bloomington, and others.

AFTERNOON SESSION.

1. Volunteer Essays, on subjects not on programme.
2. Continuation of regular Essays and Discussions.

EVENING SESSION

Essays and Discussions.

THURSDAY, MAY 13TH—MORNING SESSION.

Clinics—Conducted by Drs. W. N. Morrison, St. Louis; W. C. Dyer, Chicago: R. Mathews, Pontiac; T. L. Gilmer, Waverly, and others.

AFTERNOON SESSION.

1. Essays and Discussions until 4 o'clock.
2. Selection of place of next meeting.
3. Election of Officers.
4. Reports of Committees.
5. Miscellaneous and unfinished Business.
6. Adjournment until 7:30 P. M., or *sine die*.

